# The KOUNCIL Consortium: From Genetic Defects to Therapeutic Development for Nephronophthisis

**DOI:** 10.3389/fped.2018.00131

**Published:** 2018-05-07

**Authors:** Kirsten Y. Renkema, Rachel H. Giles, Marc R. Lilien, Philip L. Beales, Ronald Roepman, Machteld M. Oud, Heleen H. Arts, Nine V. A. M. Knoers

**Affiliations:** ^1^Department of Genetics, Center for Molecular Medicine, University Medical Center Utrecht, Utrecht University, Utrecht, Netherlands; ^2^Department of Nephrology and Hypertension, University Medical Center Utrecht, Utrecht University, Utrecht, Netherlands; ^3^Department of Pediatric Nephrology, Wilhelmina Children's Hospital, University Medical Center Utrecht, Utrecht University, Utrecht, Netherlands; ^4^UCL Great Ormond Street Institute of Child Health, London, United Kingdom; ^5^Department of Genetics, Radboud University Medical Center, Nijmegen, Netherlands; ^6^Department of Pathology and Molecular Medicine, McMaster University, Hamilton, ON, Canada

**Keywords:** cilia, pediatric kidney disease, nephronophthisis, renal ciliopathy, genetics

## Abstract

Nephronophthisis (NPH) is the most common monogenic cause of renal failure in children. Treatment options are limited to dialysis and transplantation. Therapeutics to significantly delay or prevent end-stage renal disease (ESRD) in children are currently not available. In the Dutch-Anglo KOUNCIL (**K**idney-**O**riented **UN**derstanding of correcting **CIL**iopathies) consortium, several groups and specialties united to perform scientific groundwork with the aim to develop genetic and therapeutic personalized care for NPH patients. At the start of this consortium, a genetic diagnosis for NPH was available for only 30–40% of patients, which improved to 50–60% during the course of the 4-year KOUNCIL project. Other major accomplishments of the consortium were (1) the establishment of a Dutch renal ciliopathy patient database with genotype and phenotype data; (2) composition of a proteomics-based integrated network of protein modules disrupted in NPH; (3) the development of non-invasive, urine-based assays that allow functional assessment of genomic variants in NPH and of therapeutic efficiency of drugs; and (4) chemical screening toward the identification of compounds that delay or prevent disease progression in NPH, which resulted in four potential medical interventions for NPH. In conclusion, the KOUNCIL consortium effectively channeled complementary approaches to broaden our understanding of NPH pathogenesis, resulted in 54 publications, improvement of genome diagnostics for NPH patients, awareness in the nephrology and clinical genetics communities for NPH, and new avenues for patient management.

## Introduction

Nephronophthisis (NPH; OMIM Phenotypic Series PS256100) is an autosomal recessive, genetically heterogeneous disorder that results in chronic renal disease in children and young adults (1). NPH is initially characterized by polydipsia and polyuria. Progression of the disease is characterized by renal interstitial fibrosis, tubular basement membrane disruption, and—in a subset of cases—renal cyst formation, eventually leading to renal failure. NPH can occur as an isolated disorder but is also often accompanied by a variety of extrarenal manifestations such as in Senior-Løken-, Joubert-, Bardet-Biedl-, Meckel-Gruber-, Jeune-, Short-Rib-Polydactyly-, and Sensenbrenner syndromes. These disorders overlap phenotypically, as well as genetically and functionally. All are thought to result from defective ciliary signaling, and are classified as renal ciliopathies or nephronophthisis-related ciliopathies (NPH-RCs). Although NPH patients are usually diagnosed when renal failure has developed, NPH patients typically experience a 5–10-year period between diagnosis and renal replacement therapy, which offers a therapeutic window of opportunity. Currently, no medical interventions are available for NPH patients. We anticipate that the identified molecular defect in each patient will ultimately steer the development and use of personalized therapies (2).

Gene identification is essential for development of personalized therapies. However, genetic causes of NPH are unknown in ~50% of cases (3, 4). Twenty genes are known to date to be mutated in NPH. The vast majority of gene mutations results in loss-of-function of proteins that are important for ciliary architecture or regulation of signaling cascades. Cilia are the tail-like microtubule-based protrusions from the plasma membrane that reside on the apical surface of virtually every vertebrate cell, including renal tubular cells. Cilia act as the cell's antenna by sensing the extracellular environment through an array of specific receptors and transmitting a signal response to the cell that reacts upon the signal. Ciliary gene disruption can disrupt cilia-dependent signaling cascades, which may lead to apoptosis and misaligned cell division (5). This results in heterogeneous phenotypes that affect multiple organs, including kidneys (6).

Despite steady progress in gene discovery over the past decade, a significant number of genes mutated in NPH still await identification because a significant proportion of NPH cases remains unexplained. To address this issue and advance NPH research and diagnostics collectively, our research groups united in a Dutch-Anglo consortium, funded by the Dutch Kidney Foundation. In this review, we aim to demonstrate the value of synergistic consortia in obtaining the critical mass required for patient cohort recruitment, while combining complementary expertise to work toward gene identification and targeted therapies for NPH.

## The KOUNCIL consortium

**Figure 1:**
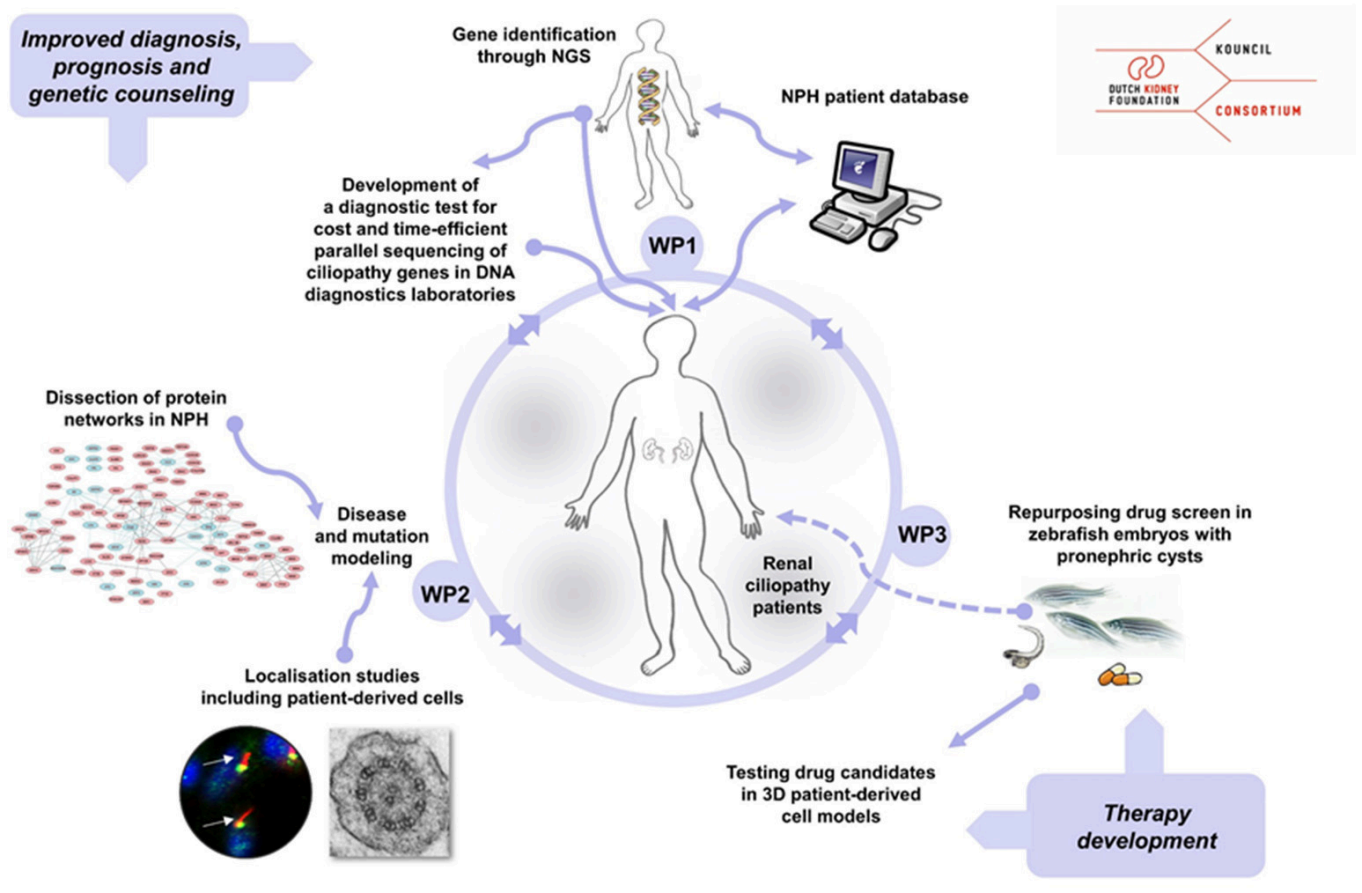
Overview of the KOUNCIL consortium work plan that aims to improve personalized diagnostics, counseling, and treatment for nephronophthisis (NPH) patients. In work package 1 (WP1), genotypes and phenotypes were extensively studied and recorded in the nephronophthisis (NPH) database. In work package 2 (WP2), NPH protein modules were identified and NPH-related genes and gene variants were functionally characterized *in vitro* and *in vivo*. Work package 3 (WP3) focused on therapy development by drug screening in zebrafish that displayed nephric cysts. NGS; next generation sequencing.

The KOUNCIL (**K**idney-**O**riented **UN**derstanding of correcting **CIL**iopathies) consortium was founded to perform scientific groundwork for the improvement of personalized care for NPH patients. Our consortium augmented the individual expertise of the collaborating workgroups toward reaching the challenging goals. The consortium included research groups with established expertise in renal ciliopathy research and diagnostics, including clinical geneticists, pediatric nephrologists, molecular biologists, cell biologists, and bioinformaticians (Figure 1). The international scientific advisory board of the Dutch Kidney Foundation reviewed and supported the consortium, and a tailored internal scientific advisory board reviewed our progress and suggested solutions to problems encountered.

We designed and conducted an integrated work plan aimed to (1) identify novel genes associated with NPH and related disorders by using next generation sequencing (NGS) approaches, (2) gain insights in genotype-phenotype relations through the setup and analysis of a renal ciliopathy database, (3) increase our understanding of the composition and function NPH-related protein modules in the ciliary proteome and explore mutational effects by state-of-the-art proteomics, and (4) initiate the development of targeted therapies to delay or prevent renal failure in NPH patients by drug screening in zebrafish and 3D culturing of cells derived from patients' urine samples.

By bringing together the three university medical centers in our project we maximized patient inclusion. This was an important aspect of our study as NPH and -related disorders are individually rare. Combining our efforts has led to significant conclusions and high impact findings. The interactions within and between the different groups and work packages have been very open and effective, and by connecting different disciplines in the KOUNCIL project, a robust workflow could be implemented in the clinic that results from this synergy. We believe that without the close and successful collaboration within the consortium, the results would have been less optimal.

## Results of KOUNCIL

### Patient recruitment, database generation, and diagnostics

**Figure 2:**
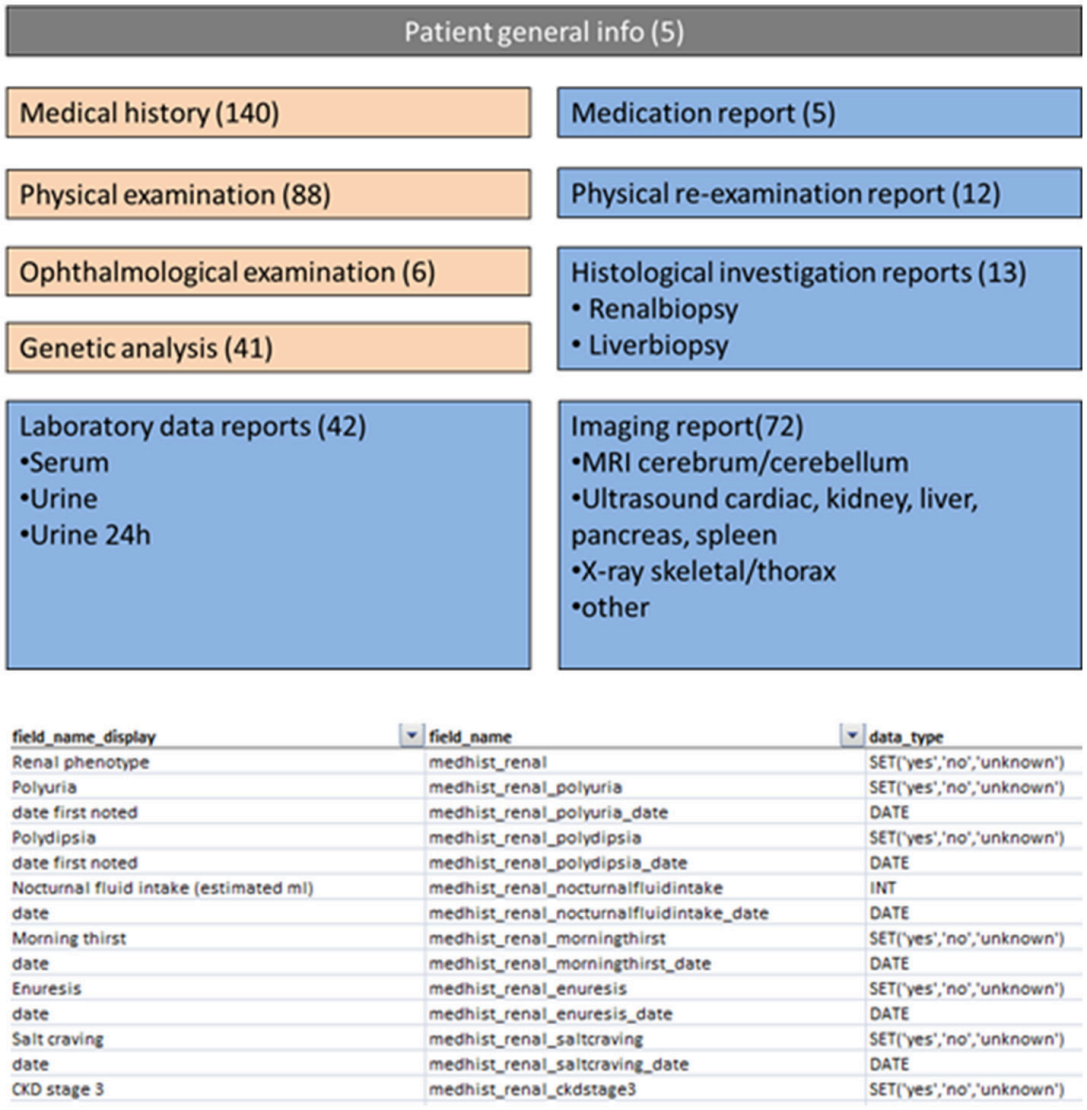
Screenshots from parts of the nephronophthisis database. Numbers indicate the number of fields attributed to a specific theme.

The success of research consortia relies heavily on patient numbers, diagnostic accuracy, and deep-phenotyping of patients. In KOUNCIL, we used the AGORA data- and biobank protocols to systematically recruit and include patients (http://www.agoraproject.nl) (7). Furthermore, we involved the professional Society for Dutch Pediatric Nephrologists and requested participation of pediatric nephrologists in recruiting patients for the renal ciliopathy database. Patients were also directly informed via the Dutch Kidney Patient Association and the KOUNCIL website (http://www.kouncil.nl). Clinical data including renal and extrarenal phenotypic information was obtained and manually curated for inclusion in the renal ciliopathy database (Figure 2). So far, 88 patients with NPH and -related disorders have been included in our coded database. Inclusion is ongoing and the database will be maintained as a resource during the following years. The database is a valuable source of clinical and genetic information, and is currently being assessed for investigations on genotype-phenotype correlations to improve early diagnosis, prognosis, and genetic counseling. Furthermore, the database offers opportunities to match with other renal ciliopathy databases such as the German language cohort Nephreg (http://www.nephreg.de), and we hope to follow up on that initiative.

We applied NGS approaches to identify causative genetic defects in 50 patients with unexplained renal ciliopathies. These methods include targeted gene panel sequencing and whole exome sequencing that facilitate the parallel analysis of multiple genes in one sequencing run in a cost- and time-efficient manner. This has led to the identification and confirmation of multiple genes for renal ciliopathy-related diseases: *WDR60* (8), *WDR34* (9), *IFT172* (10), *DCDC2* (11), *CEP120* (12), *ICK* (13), *EXTL3* (14), and *TCTEX1D2* (15). Causal variant identification in new and known genes resulted in a genetic diagnosis for 31 patients from our cohort, who had not received a prior molecular diagnosis. We have implemented renal ciliopathy-specific gene screening in our genome diagnostic divisions and ensure high sensitivity and optimal diagnostic yield by periodically updating available tests.

### Understanding functional defects underlying renal ciliopathies

**Figure 3:**
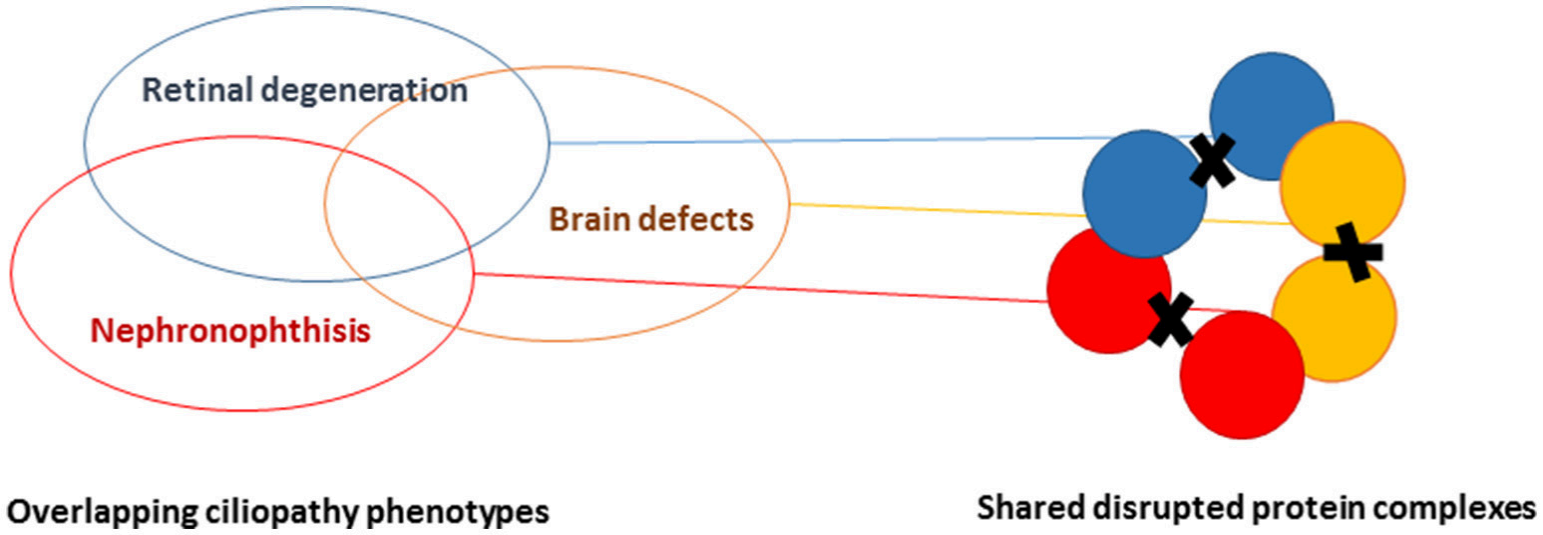
Schematic representation of understanding proteomic modules underlying ciliopathies. By performing systematic agnostic proteomic profiling of protein complexes, it is possible to distinguish and find overlap in the disrupted protein modules in ciliopathies, featuring retinal degeneration, brain malformation, and/or renal ciliopathies.

**Figure 4:**
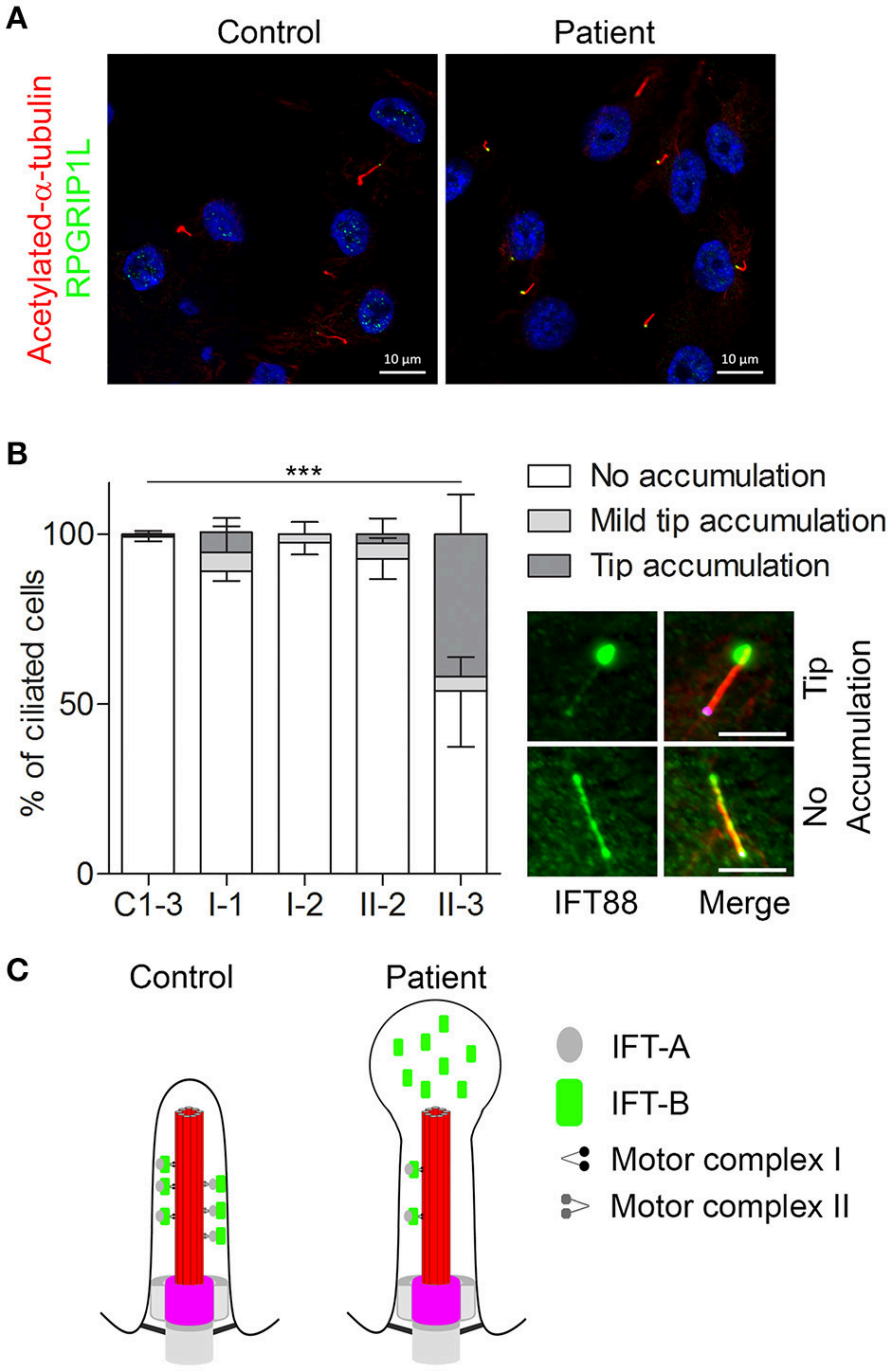
Defective retrograde transport in urine-derived cells from a Mainzer-Saldino syndrome patient. URECs derived from a patient (referred to as “patient” or “II-3”) with compound heterozygous variants in *IFT140* (one pathogenic frameshift and one missense variant of uncertain significance), were compared to URECs from three control individuals (C1-3). **(A)** URECs stained for ciliary marker acetylated-α-tubulin (red) and RPGRIP1L (green). **(B)** Analysis of retrograde transport in controls, healthy family members (I-1, I-2, and II-2) and patient (II-3) URECs. Cilia were visualized with acetylated-α-tubulin (red) and RPGRIP1L (pink). The cells were analyzed for the presence of IFT88 (green) accumulation at the ciliary tip. Patient II-3 showed a significant tip accumulation of IFT88 in 41% of the cells (Fisher's exact two-tailed test showed a *p* < 0.0001 when comparing the cells of the patient to those of the controls). Scale bar represents 5μm. **(C)** A schematic overview of the cilium in controls and in the patient with pathogenic variants in *IFT140*. The control shows normal intraflagellar transport with the IFT-B complex transporting proteins from the base of the cilium to the tip (axoneme is shown in red and the transition zone marks the base of the cilium in pink) and the IFT-A complex transporting proteins back down to the base. The image shows defective retrograde transport leading to an abnormal bulge at the ciliary tip, which is filled with IFT-B proteins. This image was adapted from Oud et al. (19). https://ciliajournal.biomedcentral.com/articles/10.1186/s13630-018-0055-2.

Cilia are regulated by defined protein complexes, some of which act as molecular machines (16). It is estimated that 1,000–2,000 proteins contribute to ciliary architecture or signaling. Through large-scale interaction assays, many of these proteins have been delineated in functional modules that are interconnected. Disrupting ciliary protein-protein interactions interferes with modules regulating cilia function. Because different ciliopathies are characterized by multiple and overlapping phenotypes, we hypothesized that there is also overlap in the molecular defects and protein modules that are disrupted in the different ciliopathies, including eye, brain, and kidney abnormalities (Figure 3) (17). Work that was performed prior to KOUNCIL suggested that proteomics-based dissection of molecular modules can relate biological mechanisms to specific genotypes in ciliopathy patients (18). Proof of principle ignited our interest to expand this approach to renal ciliopathies. Although our approach involving state-of-the art quantitative proteomics such as Stable Isotope Labeling with Amino acids in Cell culture (SILAC) combined with mass spectrometry proved technically challenging and involved multiple rounds of troubleshooting and adaptation, we were eventually successful in increasing our understanding of the ciliary protein complexes that are disturbed in NPH and allied ciliopathies (Figure 4 and unpublished data), which is indispensable for the development of targeted therapies (17, 18). Another lesson learned was that patients remain their own best model of disease, since patient cells such as urine-derived renal epithelial cells (URECs) and fibroblasts delivered the most relevant information about possible pathogenic effects of detected genomic variation and more generally, mechanisms of disease (Figure 4) (20). Thus, implementation of the developed non-invasive methods for patient-derived cell investigations has proven to be tremendously useful in diagnostics for renal diseases and therapy development.

### Advancing therapeutics for NPH

Since there is no targeted cure for NPH, research toward the development of therapies that slow renal disease progression in NPH patients is warranted. We aimed to identify drugs that delay or prevent renal cyst formation, and used zebrafish embryos with pronephric cysts for a high-throughput drug screen to achieve this goal. We first developed a robust zebrafish model for NPH, using both morpholinos and CRISPR/Cas9 gene editing technologies. A popular drug screening strategy is repurposing, whereby already FDA-approved drugs are investigated for new uses. We hypothesized that such a strategy is likely effective toward the identification of drugs that slow renal failure in NPH as it would allow a fast conversion from drug identification to actual clinical use. As such, we tested a library of FDA-approved compounds for inhibitory effects on renal cystogenesis. Two approaches were taken to screen for effective compounds. First, we performed a screen of 640 drugs in zebrafish embryos with NPH, which was a non-hypothesis driven approach. Secondly, we extensively examined 14 therapies based on known beneficial effects in polycystic kidney disease (PKD), as a candidate-selected approach. From the second approach, four individual drugs and one drug combination significantly rescued the NPH phenotype in zebrafish embryos with acceptable toxicity profiles. These drugs warrant further *in vivo* investigations for their potential in modulating NPH-progression.

To further advance therapy development, a major effort in KOUNCIL was the standardization of the use of URECs in molecular and cellular diagnostics (20). The 3D culturing of URECs resulted in clear characteristic growth patterns in NPH patients. This allowed us to adapt a non-invasive drug screening methodology in patient-derived 3D spheroids from urine. Although expansion and validation of the URECs and 3D models is ongoing, we are excited by the potential this patient-centered approach offers.

## Advances for NPH-RC patients and their families

Our project advanced medical care for kidney patients and their families. First, NGS-based methods that were implemented in DNA diagnostics at the start of our project almost doubled the diagnostic yield for NPH-RC. Secondly, NGS-based DNA diagnostics for renal ciliopathies allow clinicians to make the NPH diagnosis at a much earlier stage, which resolves the need for invasive renal biopsies for diagnostic purposes. Third, a solidified work flow has been implemented for renal ciliopathy patients in our dedicated multidisciplinary outpatient clinics for kidney diseases in the Radboud university medical center, Nijmegen, The Netherlands and the University Medical Center Utrecht, Utrecht, The Netherlands. In London, UK, multi-disciplinary clinics for Bardet-Biedl Syndrome (BBS) patients are provided. NPH-RC patients are seen by a nephrologist, ophthalmologist, geneticist, dietician, general pediatricians, endocrinologist (diabetes type II), and a clinical psychologist. The aim is to provide a “one stop” visit to ensure that patients receive specialized and expert attention and management. This should result in a major change in how NPH and BBS is managed, with a focus on diagnosis, early intervention and appropriate health management. These multidisciplinary clinics form a role model for other hospitals worldwide and will hopefully be more widely implemented in the near future.

The fourth improvement in patient care results from the setup of a renal ciliopathy database, which increased our insight into the relation between genotypes and phenotypes of NPH-RC patients. In the future, the database can be connected to other databases and converted to an interactive, international online registry. This will in turn allow for more refined insights in genotype-phenotype correlations and prognosis (6). Finally, we have developed a non-invasive protocol for research on renal cells that does not require patients (and their parents) to visit the hospital. In conclusion, KOUNCIL improved opportunities for patient management, led to advancements in the diagnostic trajectory and resulted in optimal genetic counseling for patients and their families.

## Scientific impact

KOUNCIL and KOUNCIL collaborations revealed several new renal ciliopathy genes and molecular mechanisms of disease, which significantly improved the diagnostic yield for NPH and related disorders as well as advanced knowledge of ciliopathy pathophysiology. In combination with the phenotypic characteristics sampled in our coded renal ciliopathy database, the power of genotype-phenotype correlations for NPH is just emerging and will require further prospective validation. Continued genetic screening and ongoing deep phenotyping of NPH-RC patients will further dissect these correlations, ultimately improving patient counseling. Understanding the impact of allelic variants from patients on disease manifestations demands understanding of the extensive molecular connectivity of cilium-directed pathways; to this end, we have developed a proteomics-based approach to evaluate the assembly defects of the ciliary transport particles (IFT) in a subset of ciliopathy patients. Although ciliopathies represent a class of rare pediatric diseases, through the work of KOUNCIL, we have found novel insights into the molecular mechanisms of chronic renal disease progression widespread in the general population. Preliminary work suggesting that cilia are key repressors of cellular fibrosis in the kidney, will direct and further streamline future efforts to develop therapeutic intervention with a focus on fibrosis treatment.

It should be noted that development of methodologies independent of animal experimentation are a priority for most scientific bodies today. We have generated a robust protocol growing renal cells from urine that is non-invasive to patients, yet is patient-specific, and is amenable to screening (20). We showed that functional tests in URECs and CRISPR/Cas9-derived knockdown cells can more widely clarify the pathogenicity of genetic variants of unknown significance identified in NPH-RC patients. Growing 3D spheroids from urine (“urinoids”) may also be appropriate for variant interpretation and with appropriate quality assurance measures, are potentially adaptable for diagnostic support (21).

## Conclusions

The KOUNCIL consortium, consisting of pediatric nephrologists, clinical geneticists, molecular biologists and bioinformaticians, provided a unique, coherent inter- and transdisciplinary team to combat juvenile kidney failure. The resulting five PhD theses from three centers in two countries implicated the relevance of our consortium for the next generation of renal clinicians and scientists, and will continue to have a high impact on the renal field.

This consortium aimed to improve the understanding of genetics and biology of renal ciliopathies with innovative NGS and biochemical techniques, and set the first important steps toward therapeutic approaches to delay the progressive degenerative defects in the kidney. The results of this consortium project have already significantly impacted the clinical management of NPH-RC by improvements in diagnosis, prognosis, and accuracy of genetic risk assessment. Our project has provided us with optimal preparations for the development and refinement of future personalized therapies, and is a message of hope for NPH patients for whom there is currently no satisfactory treatment.

## Author contributions

KR, RG, and HA performed the writing of the manuscript; ML, MO, and RR provided figures; RG, PB, RR, HA, and NK were work package leaders in the KOUNCIL consortium. All authors provided feedback on the revised manuscript.

### Conflict of interest statement

The authors declare that the research was conducted in the absence of any commercial or financial relationships that could be construed as a potential conflict of interest. The handling Editor declared a shared affiliation, though no other collaboration, with two of the authors: RR and MO.
